# Practical Preparation of Elastomer-Immobilized Nonclose-Packed Colloidal Photonic Crystal Films with Various Uniform Colors

**DOI:** 10.3390/polym15102294

**Published:** 2023-05-12

**Authors:** Momoko Kobori, Yuna Hirano, Mikako Tanaka, Toshimitsu Kanai

**Affiliations:** Graduate School of Engineering Science, Yokohama National University, 79-5 Tokiwadai, Hodogaya-ku, Yokohama 240-8501, Japan

**Keywords:** colloidal crystals, tunable photonic crystals, elastomers, stimuli-sensitive materials, color materials

## Abstract

Colloidal photonic crystals, which are three-dimensional periodic structures of monodisperse submicron-sized particles, are expected to be suitable for novel photonic applications and color materials. In particular, nonclose-packed colloidal photonic crystals immobilized in elastomers exhibit significant potential for use in tunable photonic applications and strain sensors that detect strain based on color change. This paper reports a practical method for preparing elastomer-immobilized nonclose-packed colloidal photonic crystal films with various uniform Bragg reflection colors using one kind of gel-immobilized nonclose-packed colloidal photonic crystal film. The degree of swelling was controlled by the mixing ratio of the precursor solutions, which used a mixture of solutions with high and low affinities for the gel film as the swelling solvent. This facilitated color tuning over a wide range, enabling the facile preparation of elastomer-immobilized nonclose-packed colloidal photonic crystal films with various uniform colors via subsequent photopolymerization. The present preparation method can contribute to the development of practical applications of elastomer-immobilized tunable colloidal photonic crystals and sensors.

## 1. Introduction

Colloidal photonic crystals are three-dimensional periodic structures of monodisperse submicron-sized particles [1,2]. They generate optical stopbands to show Bragg reflection colors, making them potentially useful in novel photonic applications and color materials [3,4,5]. In particular, nonclose-packed colloidal photonic crystals immobilized in soft polymers have received significant attention because of their high tunability. For instance, nonclose-packed colloidal photonic crystals immobilized in stimuli-responsive hydrogels can alter their optical stopband wavelength over a wide range in response to external stimuli, such as changes in pH [6,7], temperature [8,9,10], and swelling solvent [11,12,13,14]. This can be attributed to the large change in the lattice spacing of loosely packed colloidal crystals caused by the volume change of the gel. Recently, colloidal photonic crystals immobilized in elastic polymers, i.e., elastomers [15,16,17], have been developed as facilely tunable colloidal photonic crystals [18,19,20]. In contrast to gel-immobilized colloidal photonic crystals, they do not contain swelling solvents and exhibit excellent elasticity and strength; hence, the stopband wavelength can be easily altered in ambient atmosphere by applying mechanical stress. Therefore, elastomer-immobilized colloidal photonic crystals have considerable potential for use in tunable photonic applications and strain sensors that detect strain based on color change [21,22,23,24,25,26,27]. Although the preparation of elastomer-immobilized nonclose-packed colloidal photonic crystals with various uniform Bragg reflection colors is essential for their application, few studies on their practical preparation have been reported.

We previously reported that gel-immobilized nonclose-packed colloidal photonic crystal films with uniform Bragg reflection colors over several square centimeters could be prepared by flowing water suspensions of charged colloids and subsequently photopolymerizing the gelation reagent dissolved in water [28,29,30,31]. Furthermore, by replacing the water contained in the gel-immobilized colloidal photonic crystal films with an elastomer precursor solution and subsequent photopolymerization, nonclose-packed colloidal photonic crystals were successfully immobilized in the elastomer films while maintaining a uniform color [32,33]. When these films were stretched, their color changed from red to blue owing to the reduction in the lattice spacing perpendicular to the direction of the thickness, with the maximum strain reaching 120% [32]. Such color changes during extension are reversible and reproducible. Although elastomer-immobilized nonclose-packed colloidal photonic crystals with various initial colors can be prepared by changing the particle size and particle volume fraction of the colloidal suspension, this approach is impractical because of the laborious and time-consuming process of repeating the preparation from the beginning for each color.

In this study, we report a facile preparation method for elastomer-immobilized nonclose-packed colloidal photonic crystal films with different uniform colors using one kind of gel-immobilized nonclose-packed colloidal photonic crystal film. Mixtures of elastomer precursor solutions consisting of 4-hydroxybutyl acrylate (HBA) and poly(ethylene glycol) phenyl ether acrylate (PEPA), which have high and low affinities for gel-immobilized colloidal photonic crystal films, respectively, were used as swelling solvents, and the degree of swelling was controlled by varying the mixing ratio of the precursor solutions. This allowed for color tuning over a wide range, thereby facilitating the preparation of various uniform colors of elastomer-immobilized nonclose-packed colloidal photonic crystal films via subsequent photopolymerization. Furthermore, we elucidated the controllable range of the Bragg wavelength in terms of the monomer concentration in the gel-immobilized colloidal photonic crystal films.

## 2. Materials and Methods

An ion-exchange resin (AG501-X8(D), Bio-Rad, Hercules, CA, USA) was added to a suspension of monodisperse polystyrene particles with a particle diameter of 160 nm (5016 B, Thermo Fisher Scientific, Waltham, MA, USA) and gently stirred for at least two weeks to deionize the suspension. The obtained charge-stabilized colloidal crystals were centrifuged, and the supernatant was removed to obtain concentrated colloidal crystals. The gelation reagent was prepared by dissolving *N*-isopropylacrylamide (NIPAM, FUJIFILM Wako Pure Chemical Corp., Tokyo, Japan) and *N*-methylolacrylamide (NMAM, FUJIFILM Wako Pure Chemical Corp., Tokyo, Japan) monomers, *N*,*N*′-methylenebisacrylamide (BIS, FUJIFILM Wako Pure Chemical Corp., Tokyo, Japan) crosslinker, and 2,2′-azobis [2-methyl-*N*-(2-hydroxyethyl)propionamide] (VA, FUJIFILM Wako Pure Chemical Corp., Tokyo, Japan) photoinitiator in ultrapure water (Milli-Q system, Merck KGaA, Darmstadt, Germany). The gelation reagent was added to the concentrated colloidal crystals such that the concentrations of the monomers (NIPAM and NMAM), BIS, VA, and polystyrene particles were 800 mM, 40 mM, 0.35 mM, and 10.7 vol.%, respectively. The mole fraction of NIPAM in the monomers, *x* = *n*_NIPAM_/(*n*_NIPAM_ + *n*_NMAM_), was adjusted to *x* = 0.3, 0.4, 0.5, 0.6, and 0.8. Colloidal crystals containing the gelation reagent were bubbled with Ar gas for 5 min and shear-flowed into a flat quartz capillary cell (channel height: 0.1 mm; width: 9 mm; length: 50 mm) to convert the polycrystalline structure into a single crystalline structure [28,34]. The cell was then irradiated with ultraviolet (UV) light (MBRL-CUV7530, MORITEX Corporation, Saitama, Japan) for 90 min to photopolymerize the gelation reagent. The reflection spectra of the colloidal crystals at normal incidence before and after UV light irradiation were measured using a fiber spectrometer (Fastevert S-2630, Soma Optics, Ltd., Tokyo, Japan), and photographs were taken using a charge-coupled device (CCD) camera (XCD-V60CR, Sony, Tokyo, Japan). The obtained gel-immobilized colloidal crystal film was removed from the cell and cut into discs 3 mm in diameter. The discs were immersed in mixtures of elastomer precursor solutions consisting of 4-hydroxybutyl acrylate (HBA, Tokyo Chemical Industry Co., Ltd., Tokyo, Japan) and poly(ethylene glycol) phenyl ether acrylate (PEPA, Sigma-Aldrich, Saint Louis, MO, USA) with 1 wt.% photoinitiator (DAROCUR 1173, BASF, Ludwigshafen, Germany) for 24 h to replace the water contained in the gel network with the precursor solutions. The discs were sandwiched between two glass slides using two cover glasses as spacers and irradiated with UV light (MBRL-CUV7530, MORITEX Corporation, Saitama, Japan) for 10 min to photopolymerize the precursor solutions. The reflection spectra of the colloidal crystal film discs at normal incidence were measured using a fiber spectrometer (Fastevert S-2630, Soma Optics, Ltd., Tokyo, Japan). The discs were photographed using a CCD camera (XCD-V60CR, Sony, Tokyo, Japan). The refractive indices of the elastomers were measured using an Abbe refractometer (DR-A1, ATAGO Co., Ltd., Tokyo, Japan).

## 3. Results and Discussion

**Figure 1:**
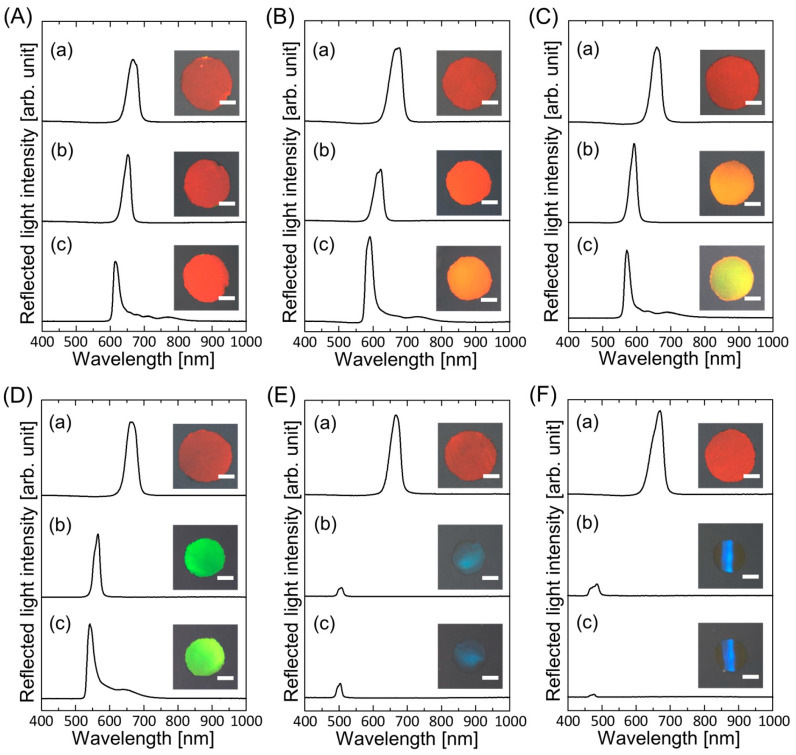
Reflection spectra and photographs of the gel-immobilized colloidal photonic crystal films with an NIPAM mole fraction of *x* = 0.5 immersed in elastomer precursor solutions with PEPA concentrations of (**A**) 0 wt.%, (**B**) 20 wt.%, (**C**) 30 wt.%, (**D**) 40 wt.%, (**E**) 60 wt.%, and (**F**) 70 wt.% at each process ((**a**) before and (**b**) after the solvent replacement and (**c**) after UV light irradiation). The length of the scale bar in the photographs is 1 mm.

Figure 1A shows the reflection spectra and photographs of the gel-immobilized colloidal photonic crystal film with an NIPAM mole fraction of *x* = 0.5 before and after the replacement of water contained in the gel film with HBA and after UV light irradiation. Before the replacement, the gel-immobilized colloidal photonic crystal film exhibited a strong peak at 667 nm in the reflection spectrum and a uniform dark red color. This peak is attributed to the Bragg reflection from the face-centered cubic (FCC) (111) lattice planes, which are parallel to the film surface [28,34]. The gel film shrank after replacement, resulting in a blue shift of the Bragg peak from 667 to 649 nm, while maintaining spectral quality. When irradiated with UV light, the elastomer precursor solution solidified to fix the nonclose-packed colloidal photonic crystals. The film size decreased after photopolymerization, causing the Bragg wavelength to decrease to 619 nm. When the gel film was immersed in an elastomer precursor solution with a PEPA concentration of 20 wt.%, it shrank more than the gel film immersed in HBA (Figure 1B). The Bragg wavelength blueshifted to 617 and 589 nm after solvent replacement and UV irradiation, respectively. Thus, the resultant elastomer-immobilized colloidal photonic crystal film exhibited a uniform orange color. As the PEPA concentration increased further, the gel film shrank, resulting in drastic color changes. As shown in Figure 1C,D, yellow and yellow-green elastomer-immobilized colloidal photonic crystal films were prepared at PEPA concentrations of 30 and 40 wt.%, respectively. At a PEPA concentration of 60 wt.%, the gel film shrank significantly, and the color turned dark blue (Figure 1E). The Bragg wavelength was significantly blueshifted to 506 nm, and the Bragg peak intensity was significantly reduced. This reduction was probably due to the decrease in refractive index contrast [35], disordering of the particle arrangement, and slight warping of the gel film caused by significant gel shrinkage. The elastomer-immobilized colloidal photonic crystal film, which maintained its spectral profile and color, was prepared via subsequent photopolymerization. When the PEPA concentration was increased to 70 wt.%, the gel film shrank further, and the resulting elastomer-immobilized colloidal photonic crystal film exhibited a blue color with significant warping (Figure 1F).

**Figure 2:**
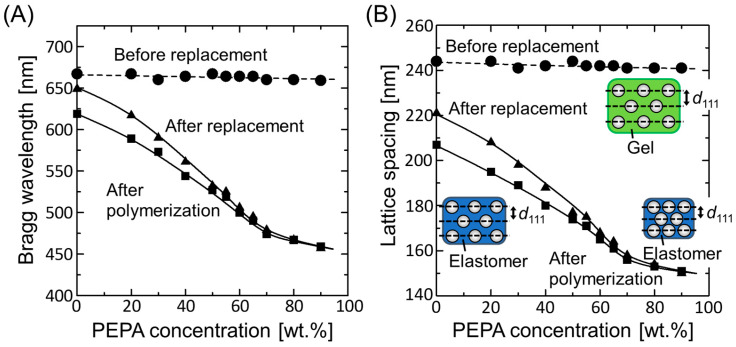
(**A**) Bragg wavelengths and (**B**) estimated FCC (111) lattice spacings of the colloidal photonic crystal films before and after solvent replacement and after photopolymerization as functions of the PEPA concentration.

Figure 2A shows the plots of the Bragg wavelengths of the colloidal photonic crystal films before and after solvent replacement and after photopolymerization as a function of the PEPA concentration, which were determined from reflection spectral measurements. As the PEPA concentration increased, the Bragg wavelength of the gel-immobilized colloidal photonic crystal film after solvent replacement decreased because the gel film shrank as the affinity between the gel network and the solvent decreased. Above a PEPA concentration of 70 wt.%, the Bragg wavelength gradually changed. The Bragg wavelengths after photopolymerization were consistently smaller than those before photopolymerization, and the difference gradually decreased with increasing PEPA concentration. These saturation behaviors suggest that the reduction in the FCC (111) lattice spacing of the colloidal crystals almost reached its limit.

The FCC (111) lattice spacing, *d*_111_, of the colloidal photonic crystal films with different PEPA concentrations in each process can be estimated from the measured Bragg wavelength, *λ*_111_, using the Bragg condition at normal incidence:

(1) $$\lambda_{111}=2n_{c}d_{111},$$

where *n*_c_ is the refractive index of colloidal photonic crystal films. *n*_c_ can be approximated as the volume-weighted average of the refractive indices of the components [32]:

(2) $$n_{c}=n_{p}\phi_{p}+n_{\mathrm{pol}}\phi_{\mathrm{pol}}+n_{\mathrm{sol}}\left(1-\left(\phi_{p}+\phi_{\mathrm{pol}}\right)\right),$$

where *n*_p_, *n*_pol_, and *n*_sol_ are the refractive indices of the polystyrene particles, polymer in the gel, and swelling solvent, respectively, and *ϕ*_p_ and *ϕ*_pol_ are the volume fractions of the particles and polymer in the film, respectively. The values of *n*_pol_ and *n*_sol_ were approximated as the volume-weighted averages of the refractive indices of the components. For the elastomer-immobilized colloidal photonic crystal film, the measured value of the refractive index of the elastomer was used as *n*_sol_. The relation between *ϕ*_p_ and *ϕ*_pol_ was determined from the masses of the particles and gelation reagent added to the suspension [10,14]. Based on geometrical considerations of a FCC structure, *ϕ*_p_ is determined using the diameter of the particles, *d*, and *d*_111_:

(3) $$\phi_{p}=\frac{2\pi }{9\sqrt{3}}{\left(\frac{d}{d_{111}}\right)}^{3}.$$

Substituting the Bragg wavelengths measured for each process into Equation (1) and using Equations (2) and (3), the FCC (111) lattice spacing was estimated, as shown in Figure 2B. The rate of change in the lattice spacing was in good agreement with the shrinking rate of the film. The lattice spacing of both the gel-immobilized colloidal photonic crystal film after solvent replacement and the resultant elastomer-immobilized colloidal photonic crystal film reached a low value of approximately 150 nm at high PEPA concentrations. This value is significantly smaller than the particle diameter (160 nm), suggesting that the lattice spacing almost reached saturation. The calculated particle volume fractions of the resultant elastomer-immobilized colloidal photonic crystal films at PEPA concentrations of 0 and 90 wt.% were 0.19 and 0.48, respectively. Because these values are much lower than the particle volume fraction of a close-packed structure (0.74), the elastomer films can be considered to have a nonclose-packed crystalline structure.

**Figure 3:**
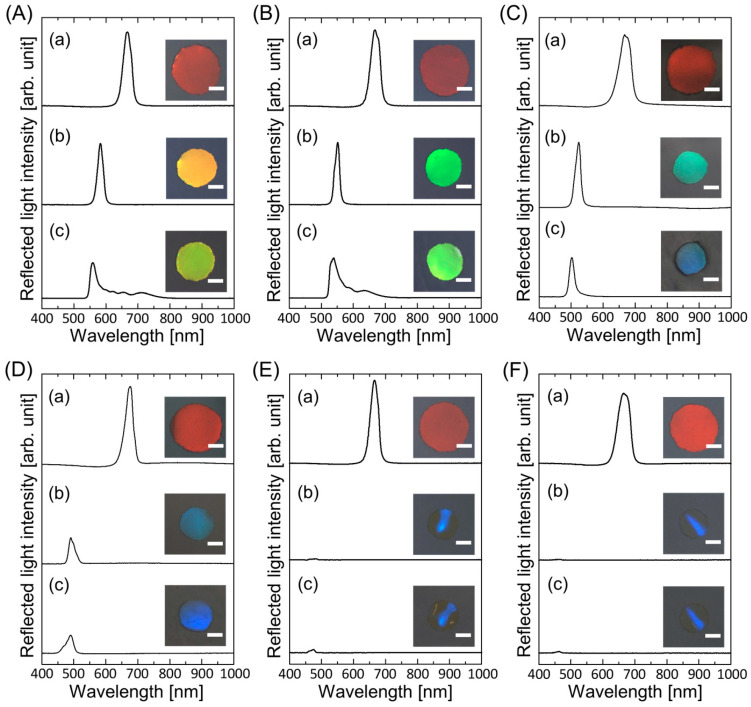
Reflection spectra and photographs of the gel-immobilized colloidal photonic crystal films with an NIPAM mole fraction of *x* = 0.4 immersed in elastomer precursor solutions with PEPA concentrations of (**A**) 0 wt.%, (**B**) 20 wt.%, (**C**) 33 wt.%, (**D**) 36 wt.%, (**E**) 50 wt.%, and (**F**) 70 wt.% at each process ((**a**) before and (**b**) after the solvent replacement and (**c**) after UV light irradiation). The length of the scale bar in the photographs is 1 mm.

Figure 3 shows the reflection spectra and photographs of the gel-immobilized colloidal photonic crystal films with an NIPAM mole fraction of *x* = 0.4 immersed in the elastomer precursor solutions with different PEPA concentrations at each process. By decreasing the NIPAM mole fraction from 0.5 to 0.4, the degree of shrinkage of the gel film and the consequent blueshift of the Bragg wavelength increased. The final elastomer-immobilized colloidal photonic crystal films exhibited yellow, yellow-green, dark blue, and blue colors at PEPA concentrations of 0, 20, 33, and 36 wt.%, as shown in Figure 3A–D, respectively. At PEPA concentrations above 50 wt.%, the resultant elastomer-immobilized colloidal photonic crystal films exhibited a blue color; however, they were significantly warped, and the Bragg reflection peak was considerably low (Figure 3E,F).

Similar results were obtained at NIPAM mole fractions of *x* = 0.6 and 0.3, as shown in Figures S1 and S2, respectively, in the Supplementary Information.

**Figure 4:**
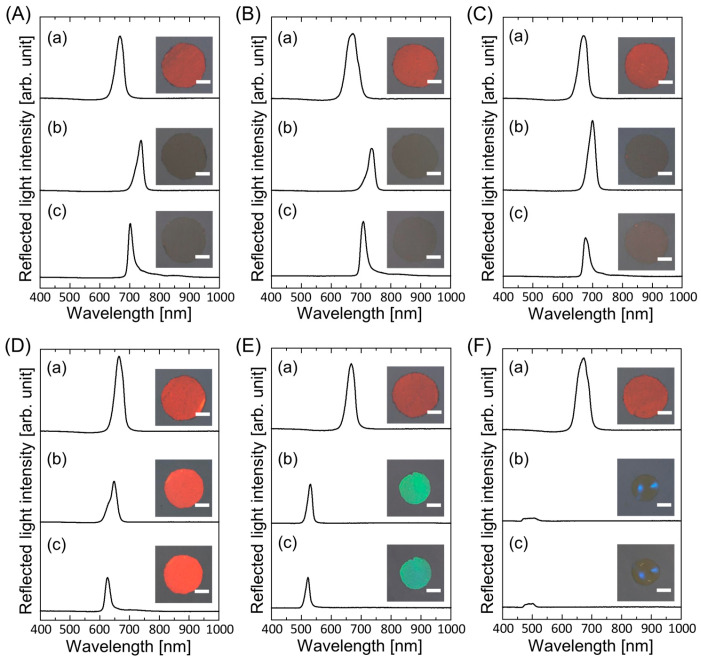
Reflection spectra and photographs of the gel-immobilized colloidal photonic crystal films with an NIPAM mole fraction of *x* = 0.8 immersed in elastomer precursor solutions with PEPA concentrations of (**A**) 0 wt.%, (**B**) 20 wt.%, (**C**) 40 wt.%, (**D**) 60 wt.%, (**E**) 80 wt.%, and (**F**) 90 wt.% at each process ((**a**) before and (**b**) after the solvent replacement and (**c**) after UV light irradiation). The length of the scale bar in the photographs is 1 mm.

When the gel-immobilized colloidal photonic crystal films with an NIPAM mole fraction of *x* = 0.8 were immersed in the elastomer precursor solutions with PEPA concentrations of 0, 20, and 40 wt.%, they surprisingly swelled more than those in water, as shown in Figure 4A–C, respectively. Thus, the Bragg peaks redshifted to more than 700 nm, and their reflection colors disappeared. Under UV irradiation, the precursor solutions solidified with slight shrinkage. On the other hand, the gel films shrank when they were immersed in elastomer precursor solutions with PEPA concentrations above 60 wt.% (Figure 4D–F). The Bragg wavelength shifted to shorter wavelengths with increasing PEPA concentration. The colors of the resultant elastomer-immobilized colloidal photonic crystal films were red, green, and blue at 60, 80, and 90 wt.%, respectively.

**Figure 5:**
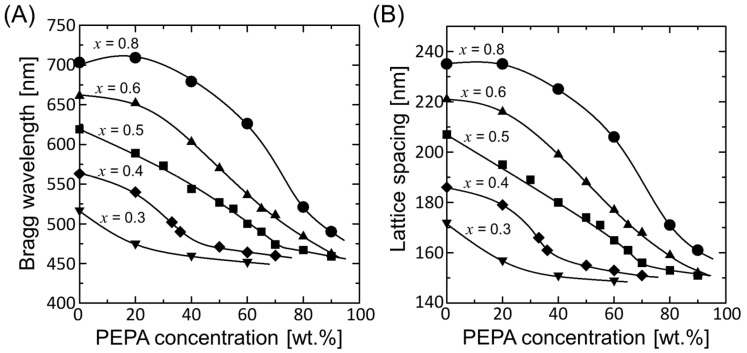
(**A**) Bragg wavelengths and (**B**) estimated FCC (111) lattice spacings of the elastomer-immobilized colloidal photonic crystal films prepared at various NIPAM mole fractions as functions of the PEPA concentration.

The measured Bragg wavelengths and estimated FCC (111) lattice spacings of the elastomer-immobilized colloidal photonic crystal films prepared at various NIPAM mole fractions are plotted as functions of the PEPA concentration in Figure 5A,B, respectively. Colloidal photonic crystal films with lower NIPAM mole fractions always exhibited smaller Bragg wavelengths and smaller lattice spacings. As the PEPA concentration increased, the Bragg wavelengths and lattice spacings decreased and then became saturated. The ultimate Bragg wavelength and lattice spacing of the most shrunken state were approximately 455 and 150 nm, respectively. The elastomer-immobilized colloidal photonic crystal film with an NIPAM mole fraction of *x* = 0.5 exhibited a linear dependence of the Bragg wavelength on the PEPA concentration in the range between 0 wt.% and 70 wt.% and showed uniform colors from red to dark blue. This indicates that the gel-immobilized colloidal photonic crystal film with an NIPAM mole fraction of *x* = 0.5 is the most suitable for the preparation of elastomer-immobilized colloidal photonic crystal films with various uniform colors because of its convenient linear tuning over a wide range.

## 4. Conclusions

Elastomer-immobilized nonclose-packed colloidal photonic crystal films with various uniform Bragg reflection colors were prepared using one kind of gel-immobilized nonclose-packed colloidal photonic crystal film. Using elastomer precursor solution mixtures composed of HBA and PEPA, which have high and low affinities for gel-immobilized colloidal photonic crystal films, respectively, as the swelling solvent, the degree of swelling was controlled by varying the mixing ratio of the precursor solutions. As the PEPA concentration increased, the Bragg wavelength of the resultant elastomer-immobilized colloidal photonic crystal film decreased. Additionally, when the NIPAM mole fraction in the gel-immobilized colloidal photonic crystal film decreased, the Bragg wavelength of the resultant elastomer-immobilized colloidal photonic crystal film decreased. The ultimate Bragg wavelength and lattice spacing of the most shrunken state were approximately 455 and 150 nm, respectively. Furthermore, the gel-immobilized colloidal photonic crystal film with an NIPAM mole fraction of *x* = 0.5 was the most suitable because the resultant Bragg wavelength exhibited a linear dependence on the PEPA concentration in the range between 0 wt.% and 70 wt.%; consequently, elastomer-immobilized colloidal photonic crystal films with uniform colors from red to dark blue were prepared. The present facile preparation method can potentially improve the practical application of elastomer-immobilized colloidal photonic crystals for tunable photonic crystals and sensors.

## Data Availability

The data presented in this study are available upon request from the corresponding author.

## Supplementary Materials

The following supporting information can be downloaded at: https://www.mdpi.com/article/10.3390/polym15102294/s1, Figure S1: Reflection spectra and photographs of the gel-immobilized colloidal photonic crystal films with an NIPAM mole fraction of *x* = 0.6 immersed in elastomer precursor solutions with PEPA concentrations of (A) 0 wt.%, (B) 20 wt.%, (C) 40 wt.%, (D) 50 wt.%, (E) 60 wt.%, (F) 65 wt.%, (G) 70 wt.%, (H) 80 wt.%, and (I) 90 wt.% at each process ((a) before and (b) after the solvent replacement and (c) after UV light irradiation). The length of the scale bar in the photographs is 1 mm; Figure S2: Reflection spectra and photographs of the gel-immobilized colloidal photonic crystal films with an NIPAM mole fraction of *x* = 0.3 immersed in elastomer precursor solutions with PEPA concentrations of (A) 0 wt.%, (B) 20 wt.%, (C) 40 wt.%, and (D) 60 wt.% at each process ((a) before and (b) after the solvent replacement and (c) after UV light irradiation). The length of the scale bar in the photographs is 1 mm.
