# Supplementary material for: Practical Preparation of Elastomer-Immobilized Nonclose-Packed Colloidal Photonic Crystal Films with Various Uniform Colors
Source: Polymers (Basel). 2023 May 12;15(10):2294. doi: 10.3390/polym15102294 (PMC10223474; doi:10.3390/polym15102294)
Supplement: Supplementary file 1 [file polymers-15-02294-s001.zip › polymers-2384132-supplementary.pdf]

Supplementary

Practical Preparation of Elastomer-Immobilized Nonclose-Packed Colloidal Photonic Crystal Films with Various Uniform Colors

Momoko Kobori, Yuna Hirano, Mikako Tanaka and Toshimitsu Kanai*

Graduate School of Engineering Science, Yokohama National University, 79-5 Tokiwadai, Hodogaya-ku, Yokohama 240-8501, Japan

*Correspondence: tkanai@ynu.ac.jp

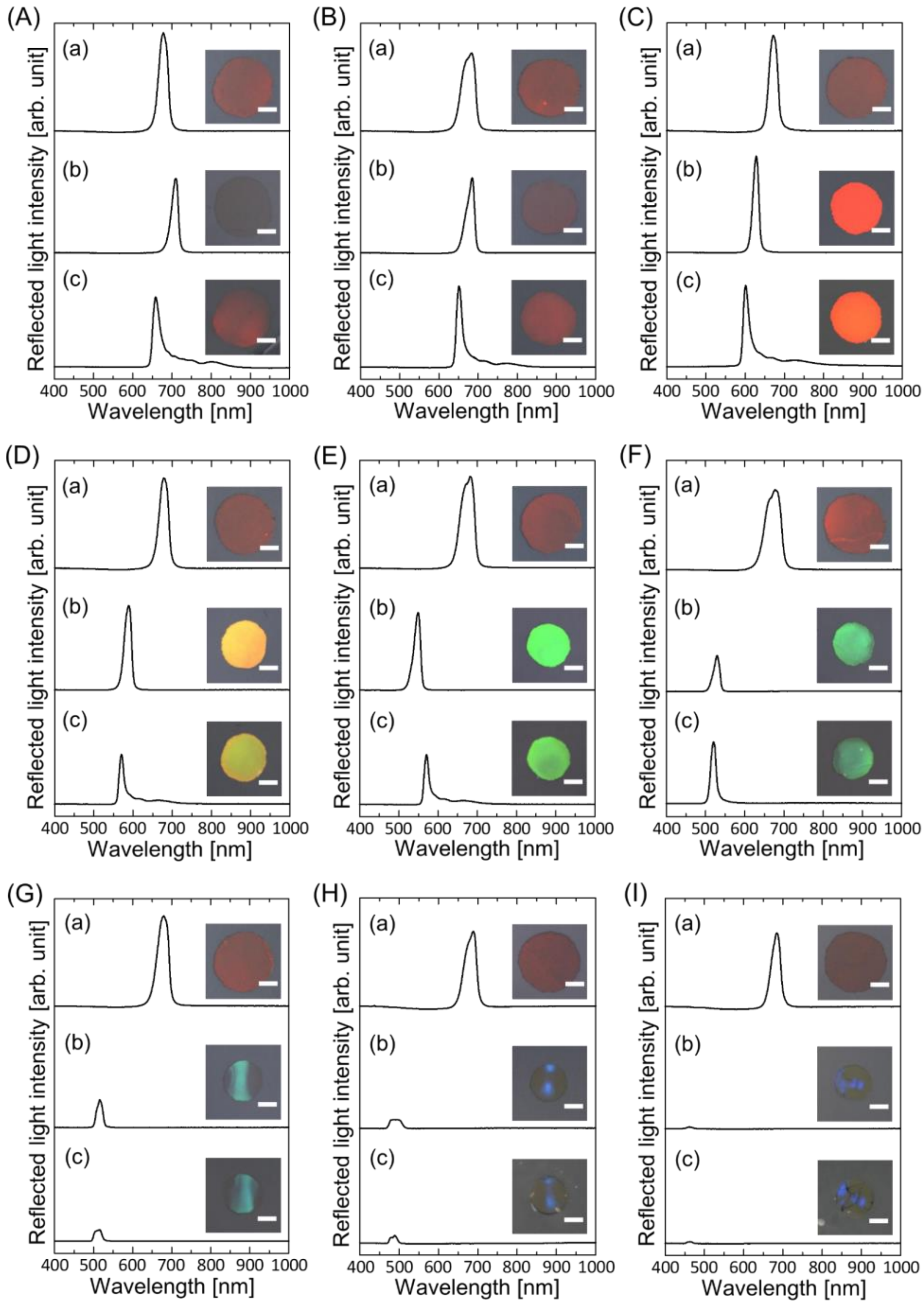

Figure S1. Reflection spectra and photographs of the gel-immobilized colloidal photonic crystal films with an NIPAM mole fraction of $x = 0.6$ immersed in elastomer precursor solutions with PEPA concentrations of (**A**) 0 wt.%, (**B**) 20 wt.%, (**C**) 40 wt.%, (**D**) 50 wt.%, (**E**) 60 wt.%, (**F**) 65 wt.%, (**G**) 70 wt.%, (H) 80 wt.%, and (I) 90 wt.% at each process ((a) before and (b) after the solvent replacement and (c) after UV light irradiation). The length of the scale bar in the photographs is 1 mm.

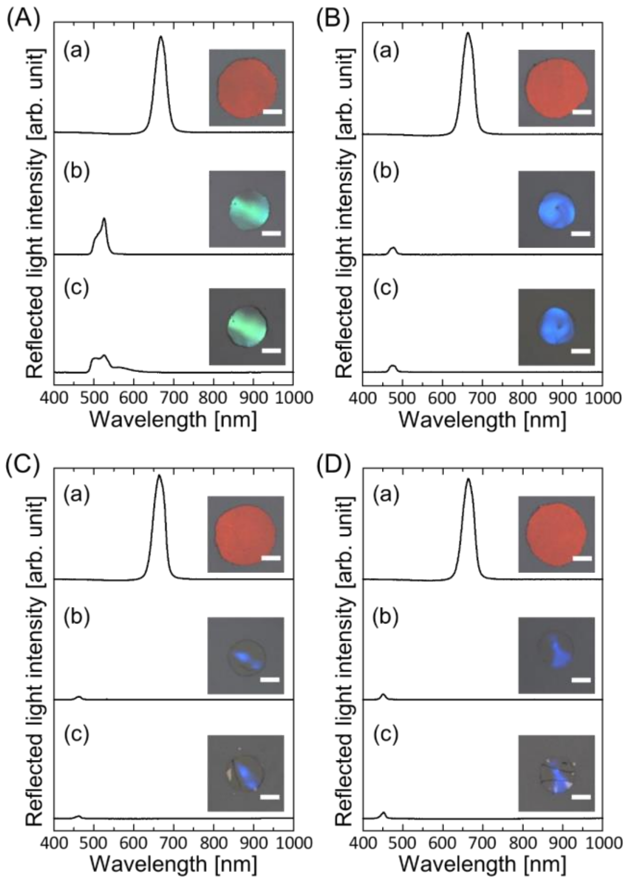

Figure S2. Reflection spectra and photographs of the gel-immobilized colloidal photonic crystal films with an NIPAM mole fraction of $x = 0.3$ immersed in elastomer precursor solutions with PEPA concentrations of (**A**) 0 wt.%, (**B**) 20 wt.%, (**C**) 40 wt.%, and (**D**) 60 wt.% at each process ((a) before and (b) after the solvent replacement and (c) after UV light irradiation). The length of the scale bar in the photographs is 1 mm.
